# Supplementary material for: Expression of Nutritional Traits in Vegetable Cowpea Grown under Various South African Agro-Ecological Conditions
Source: Plants (Basel). 2022 May 27;11(11):1422. doi: 10.3390/plants11111422 (PMC9182706; doi:10.3390/plants11111422)
Supplement: Supplementary file 1 [file plants-11-01422-s001.zip › plants-1674761-supplementary.pdf]

Supplementary tables

Table S1 Mean performance of cowpea genotypes for protein content (%) across six environments

Genotype	Mafikeng 2016	Potch 2016	Potch 2017	RPT 2016	RPT 2017	Venda 2016
Veg cowpea 1	31.23	31.34	29.94	24.57	35.21	21.24
TVU-14196	31.78	30.22	30.46	30.54	34.15	21.29
Veg cowpea 2	32.45	34.47	34.62	27.68	35.38	22.09
Meter long bean	29.57	27.80	29.96	28.12	29.23	23.19
Vigna Onb	32.85	31.92	34.4	27.75	32.54	24.78
Kisumu mix	35.22	34.42	35.6	30.04	33.33	22.66
M217	34.10	28.96	32.38	23.8	30.72	22.12
Ukaluleni	31.06	31.78	31.60	28.64	27.52	22.87
VCDC	34.12	30.58	33.50	25.69	36.04	22.70
5431	30.65	29.48	29.81	28.85	27.96	25.54
Chappy	31.70	30.56	32.67	30.06	30.21	21.27
Mamlaka	34.66	35.38	34.56	29.57	31.85	21.29
IT96D-602	31.29	29.3	30.29	25.95	30.79	23.97
98K-5301	33.35	30.81	30.38	26.95	33.44	22.36
ITOOK-1060	32.78	27.09	34.83	27.22	32.04	24.38
Grand mean	32.45	30.94	32.33	27.70	32.03	22.78
LSD	3.222	5.071	3.749	2.959	3.063	5.081

Potch = Potchefstroom, RPT = Roodeplaat, LSD = Least significant difference, VCDC = Vegetable cowpea dakama cream

Table S2 Mean performance of cowpea genotypes for Fe ($mg.100.g^{-1}$) concentrations across six environments

Genotype	Mafikeng 2016	Potch 2016	Potch 2017	RPT 2016	RPT 2017	Venda 2016
Veg cowpea 1	35.29	24.29	69.60	111.58	61.80	26.07
TVU-14196	45.24	20.59	64.30	66.59	67.40	27.68
Veg cowpea 2	28.87	21.89	68.00	48.87	153.30	44.68
Meter long bean	23.50	22.62	78.80	66.97	187.90	28.48
Vigna Onb	25.13	16.79	76.90	39.44	109.40	30.17
Kisumu mix	17.77	18.31	33.10	28.49	68.00	33.00
M217	30.80	27.27	59.70	86.40	135.60	20.05
Ukaluleni	35.78	18.98	72.20	44.04	67.00	29.06
VCDC	25.39	19.49	71.00	165.56	90.40	42.41
5431	25.52	23.79	129.40	111.35	66.20	23.73
Chappy	27.36	25.33	94.40	41.65	101.10	37.71
Mamlaka	35.47	17.17	58.20	46.60	62.40	27.21
IT96D-602	25.68	20.23	41.40	101.19	70.10	20.05
98K-5301	33.52	17.62	72.80	56.33	75.70	22.95
ITOOK-1060	41.61	18.64	62.00	41.43	60.4	29.88
Grand mean	30.46	20.87	70.10	70.43	91.80	29.54
LSD	3.725	3.993	38.85	7.333	76.87	21.878

Potch = Potchefstroom, RPT = Roodeplaat, LSD = Least significant difference, VCDC = Vegetable cowpea dakama cream

Table S3 Mean performance of cowpea genotypes for Zn (mg.100.g^{-1}) concentrations across six environments

Genotype	Mafikeng 2016	Potch 2016	Potch 2017	RPT 2016	RPT 2017	Venda 2016
Veg cowpea 1	5.30	3.07	2.723	3.04	6.17	4.01
TVU-14196	5.94	3.03	3.749	3.52	5.53	4.55
Veg cowpea 2	5.98	2.70	3.583	3.44	6.48	3.93
Meter long bean	7.00	2.86	3.846	3.97	6.54	3.97
Vigna Onb	6.14	2.53	3.462	3.01	6.19	3.88
Kisumu mix	5.01	2.85	3.734	3.45	5.83	3.96
M217	5.08	3.43	3.018	3.43	4.68	4.34
Ukaluleni	5.03	2.48	3.087	4.08	6.36	3.38
VCDC	6.40	3.33	3.566	3.75	6.39	3.86
5431	5.12	2.90	2.963	3.87	7.34	3.62
Chappy	5.13	2.13	3.697	4.13	5.61	4.17
Mamlaka	5.73	2.69	3.315	3.75	6.35	3.5
IT96D-602	5.51	2.82	3.436	3.29	5.96	4.46
98K-5301	5.57	3.57	3.014	3.27	6.01	3.68
ITOOK-1060	5.45	2.92	3.871	4.08	6.36	3.69
Grand mean	5.63	2.89	3.404	3.61	6.12	3.93
LSD	1.099	0.752	0.8653	0.634	1.481	0.76

Potch = Potchefstroom, RPT = Roodeplaat, LSD = Least significant difference, VCDC = Vegetable cowpea dakama cream

Table S4 Mean performance of cowpea genotypes for Mn (mg.100.g^{-1}) concentrations across six environments

Genotype	Mafikeng 2016	Potch 2016	Potch 2017	RPT 2016	RPT 2017	Venda 2016
Veg cowpea 1	17.73	24.00	11.69	22.66	11.54	23.11
TVU-14196	23.10	19.48	12.45	19.41	12.13	21.51
Veg cowpea 2	19.31	16.39	11.33	16.70	12.29	24.25
Meter long bean	21.76	26.10	15.75	20.69	13.76	19.71
Vigna Onb	20.18	17.38	12.23	15.87	13.00	22.39
Kisumu mix	16.63	12.76	8.62	15.48	11.69	21.24
M217	22.20	20.41	12.94	18.27	11.39	19.86
Ukaluleni	20.89	14.70	12.30	17.82	13.88	19.37
VCDC	17.36	16.90	12.16	17.09	11.20	23.33
5431	23.76	22.39	14.99	16.09	11.45	21.85
Chappy	19.12	14.99	12.95	14.54	13.31	24.45
Mamlaka	21.55	15.60	11.56	16.59	12.17	25.84
IT96D-602	21.42	18.12	10.85	14.27	11.30	14.43
98K-5301	24.25	15.74	14.98	19.21	13.13	24.90
ITOOK-1060	23.13	13.89	10.77	14.26	11.63	21.00
Grand mean	20.83	17.92	12.37	17.26	12.26	21.82
LSD	3.863	3.137	2.089	2.881	2.881	8.993

Potch = Potchefstroom, RPT = Roodeplaat, LSD = Least significant difference, VCDC = Vegetable cowpea dakama cream
